# Is the distal radioulnar variance useful for identification of radial head prosthesis overlengthening? A cadaver study

**DOI:** 10.1186/s40001-016-0196-y

**Published:** 2016-03-21

**Authors:** Kilian Wegmann, Wei Zhang, Volker Strauss, Christian Ries, Lars P. Müller, Klaus J. Burkhart

**Affiliations:** Center for Orthopedic and Trauma Surgery, University Medical Center, Josef-Stelzmann-Str.9, 50924 Cologne, Germany; Cologne Center for Musculoskeletal Biomechanics, Medical Faculty, University of Cologne, Josef-Stelzmann-Str.9, 50924 Cologne, Germany; Arcus Klinik , Raststatter Straße 17-19, 75179 Pforzheim, Germany

**Keywords:** Forearm rotation, Overlengthening, Prosthetic replacement, Radial head, Ulnar variance, Multipolar prosthesis

## Abstract

**Background:**

Overlengthening by radial head prosthetic replacement leads to insufficient functionality and increased capitellar wear. It has been shown that in monopolar prostheses, the radial overlengthening by an overstuffed prosthesis leads to significant differences in the distal radioulnar variance at the wrist. This study evaluated ipsilateral ulnar variance as a predictor for overlengthening after implantation of a multipolar prosthesis.

**Methods:**

The radial heads of seven fresh frozen upper extremities were resected and a multipolar radial head prosthesis was implanted. Thereafter, the native radioulnar variance at the wrist was documented via fluoroscopy. The alignment of the distal radioulnar joint in neutral, pronated and supinated rotational positions of the forearm was recorded fluoroscopically, and digital image analysis was performed regarding radioulnar shifting.

**Results:**

Statistical analysis of the difference between native height and the manipulated states did not show consistent significant differences with stepwise overlengthening of +1.5, +3, +4.5 and +6 mm and with respect to rotational position of the forearm (*p* > 0.05). Interclass correlation coefficients showed excellent interobserver reliability (ICC 96 %), as did tests for intraobserver reliability (ICC 98–99 %).

**Conclusions:**

No consistent influence of overlengthening on the alignment of the radius and ulna at the distal radioulnar joint was found after sequential overlengthening with a multipolar prosthesis. Maybe the ligamentous structures of the forearm prevent significant longitudinal dislocation of the radius, as the multipolar prosthesis gives way by at the radiocapitellar joint. According to the data of the present study, the ipsilateral wrist is not useful in diagnosing overlengthening of the radial column in multipolar prosthetic replacement of the radial head—in contrast to the reported results with monopolar prostheses.

## Background

Prosthetic replacement of the radial head in case of severe arthritis or in unreconstructable fractures can lead to good short- and mid-term results [1–8]. As adequate function of the implant relies on correct size and position of the implant, the height of the osteotomy plays an important role as it determines the final radial length. Overstuffed implants or insufficient resection of bone from the radial neck lead to overlengthening of the radial column and therefore impede physiologic kinematics and endanger good clinical results [9–11]. This “radial overlengthening” may lead to loss of range of motion of the elbow joint and increased joint contact forces in the radio-humeral joint with early erosion of capitulum cartilage [5, 12]. Diagnosis of prosthetic radial head overlengthening is difficult, and its management is challenging [13]. Intraoperatively, due to limited joint exposure the correct length of the radial column is difficult to choose. Thus, several authors have implemented techniques to facilitate choosing correct implant sizes and positions and correct osteotomy height intraoperatively, or to diagnose overlengthening postoperatively [14–16].

Nevertheless, the need for reliable tools to visualize overlengthening intraoperatively remains. Recently, Moon et al. have published an experimental study in which artificial radial overlengthening led to significant changes at the distal radioulnar joint with distalization of the radius [17]. To our knowledge, no study has yet evaluated an intraoperative test based on radiographic images of the wrist to diagnose radial overlengthening by a multipolar prosthesis.

We hypothesized that radioulnar variance—the relative lengths of the distal articular surfaces of the radius and ulna—can be used to identify overlengthening.

## Methods

### Specimen preparation

Seven fresh frozen upper extremities disarticulated at the shoulder joint were collected from voluntary body donors. The average age of donors was 87.0 years (range 80–104). The specimens showed adequate range of motion and ligamentous stability.

The study was approved by the local institutional review board.

### Prosthesis

The CRF-II©-Prosthesis, Floating Radial Head Prosthesis Tornier, Saint-Ismier, France, was used.

### Dissection and implantation technique

**Fig. 1:**
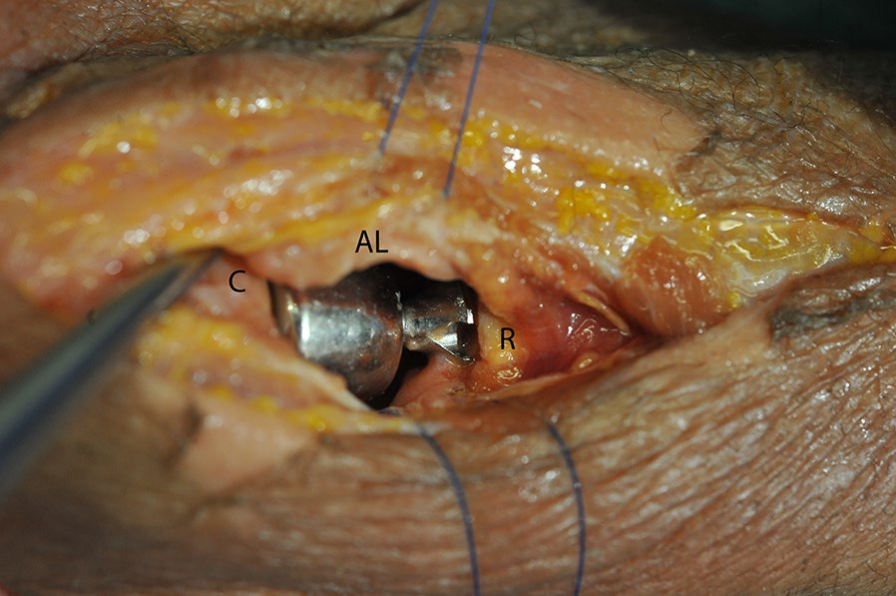
A specimen after implantation of the multipolar prosthesis in native height is shown. A Kocher approach has been used. *C* capitulum; *AL* annular ligament; *R* radius

**Fig. 2:**
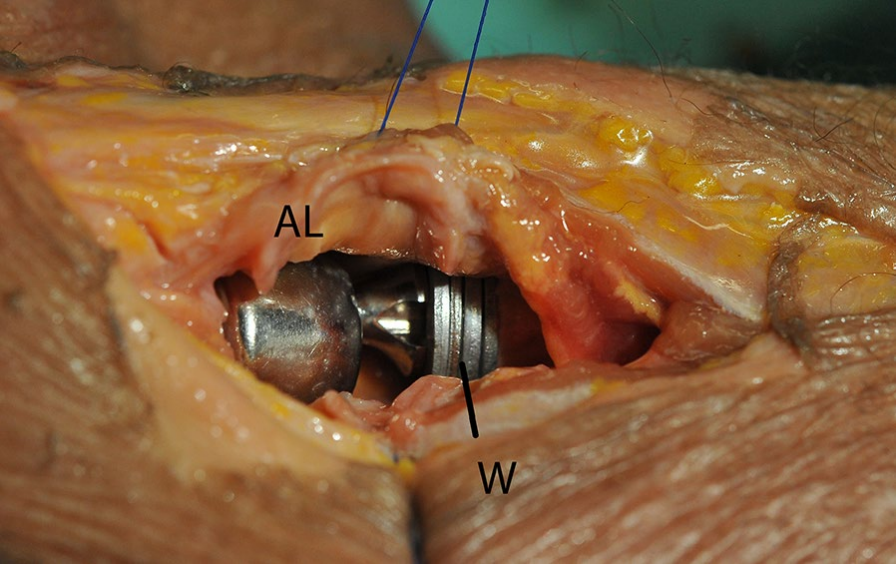
A specimen after implantation of the multipolar prosthesis in overstuffing of 6 mm with use of 4 metal washers is shown. *AL* annular ligament; *W* washers

After complete thawing of the specimens overnight, the standard posterolateral approach to the radial head described by Kocher was performed [18]. The level of the rim of the healthy radial head in relation to the proximal radioulnar joint was marked with diathermia at the proximal ulna to be able to restore the exact native height with the prosthesis. After that, the radial neck was cut by a transverse osteotomy distally to the ulnar facet with an oscillating saw. The implantation of the prosthesis aimed to achieve the native height, which was marked at the proximal ulna before osteotomy (Figs. 1, 2). The capsule and collateral ligament were closed by sutures to achieve a stable elbow joint.

**Fig. 3:**
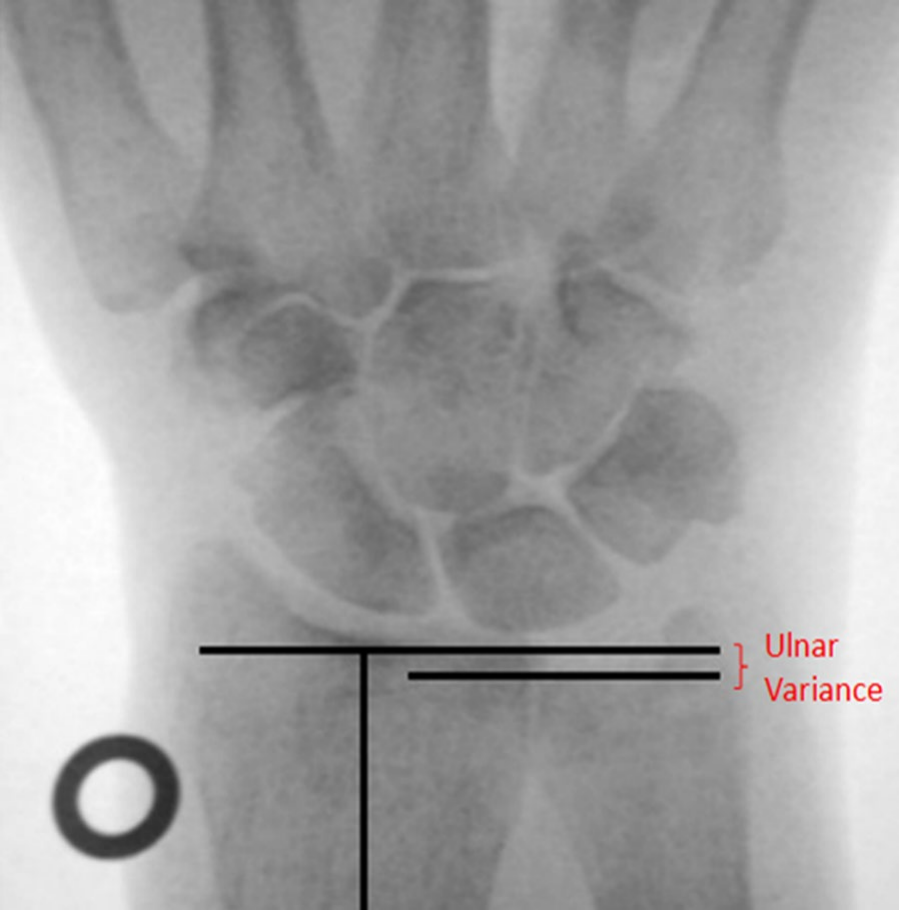
Illustration of the measuring technique that was conducted to display ulnar variance. Metal washer to the left of the image as a referencing tool

The specimens were positioned on an examination table in neutral rotation with the elbow joint flexed at 45º and the dorsal hand facing upwards. Orthogonal, dorsopalmar images of the wrist were taken with the C-arm radiographic unit. The C-arm unit was set in a fixed position in relation to the examination table and the specimens, to achieve reproducible radiographs. A metal washer was placed next to the specimens, acting as a referencing tool for the digital analyses (Fig. 3).

We simulated overlengthening by adding metal washers to the stem of the implant to modulate the length of radial head prosthesis to achieve overlengthening of +1.5, +3, +4.5 and +6 mm. Dorsopalmar radiographs of the wrist were taken again at each step of overlengthening to document radioulnar variance.

### Digital analysis of the images

The radiographic images were stored as jpeg files. Image-Pro Plus© image analysis software (Version 6.0.0.260, MediaCybernetics Inc., USA) was used for the digital measurement of radioulnar variance and the changes induced by overlengthening. First, the longitudinal axis of the radius was marked. Then, a line perpendicular to that longitudinal axis was drawn through the distal ulnar aspect of the radius. The radioulnar variance was determined by measuring the distance between this line and the distal cortical rim of the ulna (Fig. 1). To assess intra- and interobserver reliability, each measurement was performed three times by two independent observers (V.S. and W.Z.) who were otherwise blinded regarding the degree of overlengthening.

### Statistical analysis

The distribution of normality of the data was analysed by Kolmogorov–Smirnoff/Shapiro–Wilk test and graphical illustration. Median, mean, minimum and maximum values and standard deviations were calculated. The relative changes of radioulnar variance initially and after each step of overlengthening were computed, and the significance of differences between these values was evaluated with the Wilcoxon test for not-normally distributed data. The confidence interval was set at <0.05. To evaluate inter- and intrarater reliability, the intraclass correlation coefficients (ICCs) were computed using SPSS 19 software© (SPSS Inc., Chicago, Il, USA), and 95 % confidence intervals (CIs) were calculated.

## Results

**Fig. 4:**
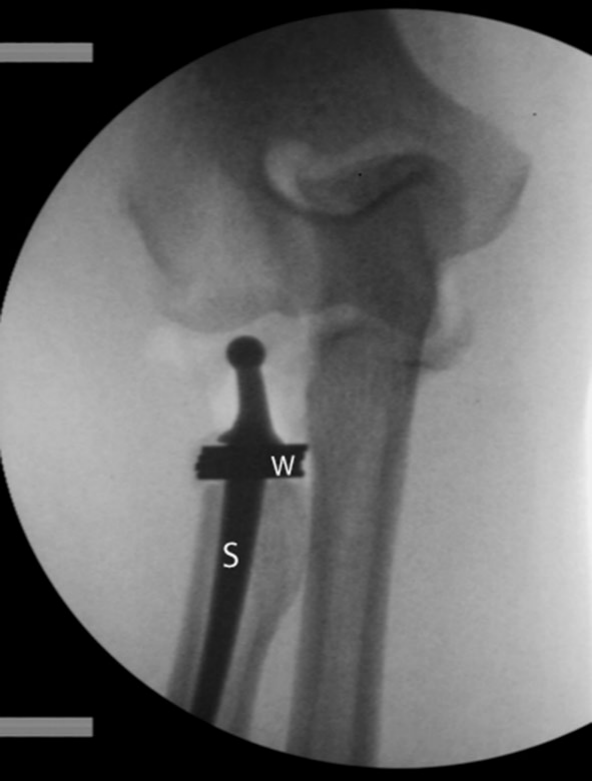
X-ray image, showing a multipolar prosthesis with an artificial overlengthening of +6 mm. The head of the prosthesis is radiolucent, as a trial head component was used. *S* radial shaft; *W* metal washers

**Fig. 5:**
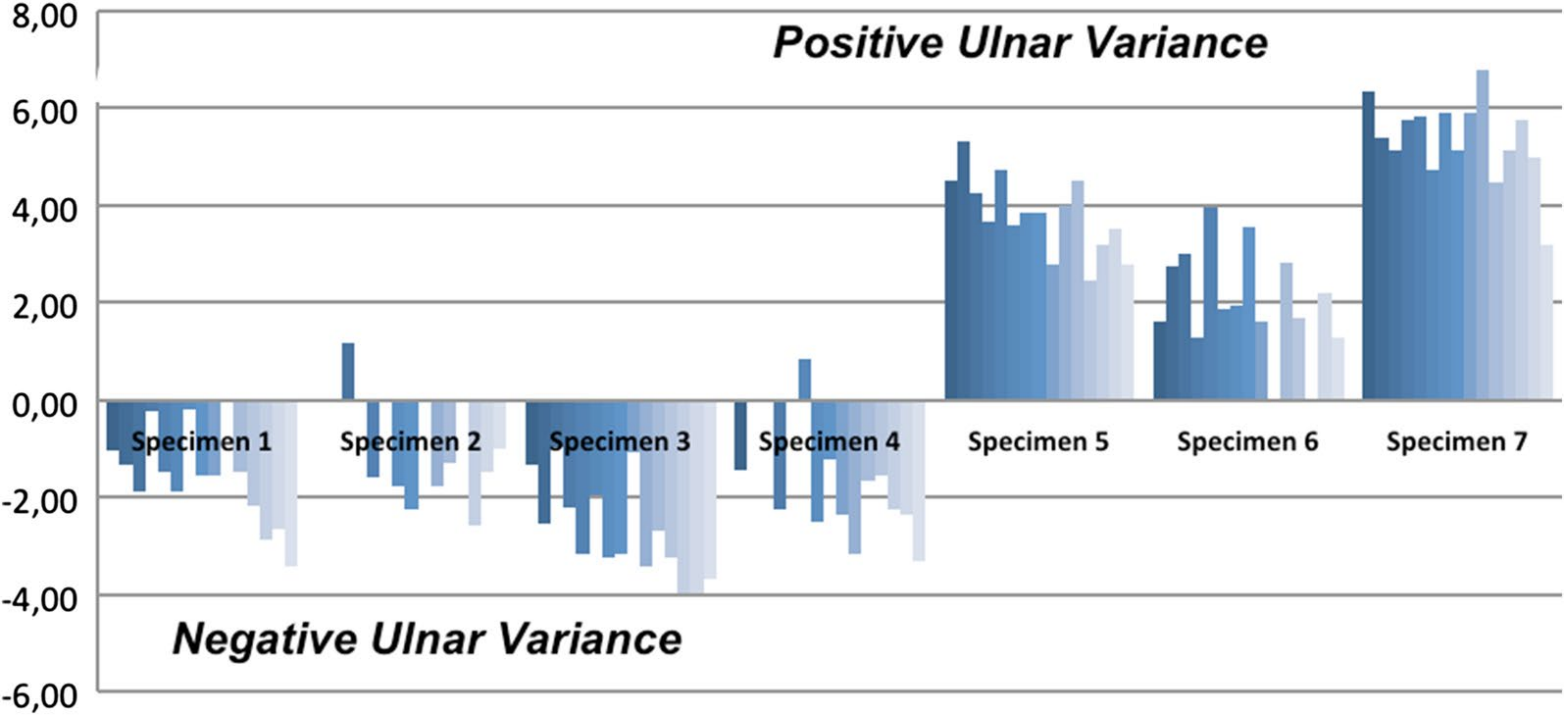
Absolute values of the ulnar variance after stepwise overlengthening with bipolar prostheses. The *dark blue bars* starting at the left represent the native state. The *white bars* to the right of each specimen display the values for the +6 mm overlengthening

**Fig. 6:**
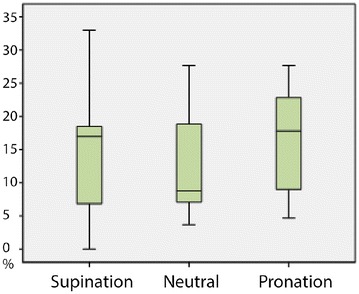
*Box plot* representing the values of the rotational position of the forearm on ulnar variance. It shows the overall influence of supination, neutral position and pronation. The inter-quartile range of the three *plots* is similar; the height does not differ significantly, supporting the fact that rotational position does not have a marked influence on the radioulnar variance in the presented setting

The data were not normally distributed (Kolmogorov–Smirnov/Shapiro–Wilk *p* < 0.01). The results for the absolute values of the radioulnar variance with the multipolar prosthesis are shown in Fig. 4. In the illustration, a trend is visible: overlengthening leads to increased negative radioulnar variance, or a decrease of positive radioulnar variance. For simplification, the values are presented as figure and not as a table. However, the analysis of the differences between the native height and the overlengthened states did not show statistically significant differences with stepwise overlengthening of +1.5, +3, +4 and +6 mm (*p* > 0.05) (Figs. 5 and 6).

### Reliability of measurements

The intraclass correlation coefficients showed excellent interobserver reliability for the average values of all three measurements that were done by the two observers (ICC = 96 %; CI 95 % = 0.95–0.97) (Fig. 6).

Intraobserver reliability was also excellent for both observers (observer 1, ICC = 98 %, CI 95 % = 0.98–0.99; observer 2, ICC = 99 %, CI 95 % = 0.97–0.98).

## Discussion

Overlengthening of the radial column with prosthetic replacement of the radial head restricts good clinical outcome. We hypothesized that radioulnar variance can assist in the identification of radial overlengthening already intraoperatively using a fluoroscopic device. However, according to the present data there was no statistical difference in radioulnar variance by stepwise overstuffing of a multipolar radial head prosthesis.

Several authors have introduced clinical or radiological techniques to facilitate deciding the correct length of the radial column for the implantation of a radial head prosthesis [14–16, 19]. Yet, other authors found some of the presented techniques to be unreliable [20, 21].

Moon et al. presented an experimental study in which they found significant differences in the radioulnar variance of the wrist after sequential overlengthening of the radial column with a monopolar prosthesis [17]. They tested the specimens in full extension and neutral position. In the presented figures, translation of the radius is obvious.

It was reasonable to expect either stepwise reduction of negative radioulnar variance at the distal radioulnar joint or an increment of positive radioulnar variance in our setup as well. The biomechanics of the elbow and wrist joint are closely related and force loaded onto the wrist travels to the elbow joint via radius and ulna [22, 23]. The interosseous membrane transfers load from the radius to the ulna. This is made possible by the run of the main fibres of the membrane ascending from the ulna to the radius, which prevent proximalisation of the radius. As the fibres get tensed by loading to the radius, they transfer force in a proximal direction onto the ulna [24]. However, there are also fibres preventing distalization of the radius against the ulna, which are mostly localised at the proximal and distal part of the forearm [25, 26]. It can be assumed that these ligamentous interactions play a significant role in the impact of overlengthening on the alignment of the proximal and distal radioulnar joints. Lanting and colleagues found that the tension within the interosseous membrane of the forearm decreased by increasing the radial head implant length [27]. Lanting et al. also found increased radiocapitellar contact pressures with increased length of the radial head implant. The pressure onto the capitulum exerted by overlengthening, leading to thrust towards distalization of the radius, might be neutralised by compensatory movements of the highly flexible multipolar design. A possible mechanism might increase angulation by anterior tilt of the mobile head component. This leads to dorsal subluxation of the shaft. Yian and colleagues found multipolar prostheses to allow compensatory movements within the radiocapitellar joint [28]. Monopolar systems in contrast do not allow compensatory movements as the shaft is amotile to the head component. This could be the reason why in the study of Moon and colleagues more distinct differences were found by overstuffing of the radial column on the radioulnar variance [17].

The presented study has limitations. Cadaver studies are limited by availability, and only seven specimens could be included. The trend of the data shows a possible relevant difference. Maybe with a higher number of specimens the data would have gained significance. Still, a marked increase in specimens might have been necessary, as the data showed high standard deviation and variance.

Also study results depend on the biomechanical properties of the used specimens. In this in vitro cadaveric study, the average age of the specimens was at 87.0 years. It remains to be confirmed whether the results are also valuable in younger patients.

Another limitation is the fact that the measurements were done in 45° of flexion in the elbow joint. According to the study of Fu and colleagues, the highest amount of longitudinal translation of radius versus ulna takes place at full flexion or full extension at the elbow joint [29]. Still, we chose neutral rotation in 45° of flexion, as it is a position of the upper extremity that is easiest to achieve during intraoperative conditions with fluoroscopy, and we aimed to develop a test that is applicable intraoperatively.

The study also did not refer to the situation in total elbow replacement. In total elbow, overlengthening of the radial column has not been prime topic of discussion. However, total elbow systems featuring the replacement of the radial head come along with cutting devices that orientate on the ulnar component. Thus, overlengthening of the radial column should not be of relevance in total elbow. Moreover, within the study we used specimens without clinical signs of preexisting radioulnar pathologies, like instability or obvious structural damage. Clearly, when implanting radial head prostheses for fracture treatment and concomitant injury to the distal radioulnar joint is suspected, the radioulnar variance cannot be used as a guide for overlengthening at all.

## Conclusions

According to the present data, radioulnar variance clearly is not suitable to identify overlengthening of the radial column by multipolar radial head prosthesis. Therefore, radioulnar variance should not be used as a clinical tool to assess overlengthening. The present study cannot explain the mechanisms of whether the multipolar head of the prosthetic component is causative, as radioulnar variance has been proven to be a sufficient indicator of overlengthening by monopolar radial head prostheses.
